# Plant Volatile Compounds of the Invasive Alligatorweed, *Alternanthera philoxeroides* (Mart.) Griseb, Infested by *Agasicles hygrophila* Selman and Vogt (Coleoptera: Chrysomelidae)

**DOI:** 10.3390/life12081257

**Published:** 2022-08-17

**Authors:** Meng-Zhu Shi, Jian-Yu Li, Yan-Ting Chen, Ling Fang, Hang Wei, Jian-Wei Fu

**Affiliations:** 1 Institute of Quality Standards & Testing Technology for Agro-Products, Fujian Key Laboratory of Agro-Products Quality and Safety, Fujian Academy of Agricultural Sciences, Fuzhou 350001, China; 2 Institute of Plant Protection, Fujian Key Laboratory for Monitoring and Integrated Management of Crop Pests, Fujian Engineering Research Center for Green Pest Management, Fujian Academy of Agriculture Sciences, Fuzhou 350013, China

**Keywords:** *Agasicles hygrophila*, *Alternanthera philoxeroides*, volatiles, herbivore-induced plant volatiles

## Abstract

Plants release a variety of volatiles and herbivore-induced plant volatiles (HIPVs) after being damaged by herbivorous insects, which play multiple roles in the interactions with other plants and insects. *Agasicles hygrophila* Selman and Vogt (Coleoptera: Chrysomelidae) is a monophagous natural enemy and an effective biocontrol agent for *Alternanthera philoxeroides* (Mart.) Griseb. Here, we reported differences among the volatiles of *A. philoxeroides* by solid phase microextraction (SPME) using a gas chromatography-mass spectrometer (GC-MS). We compared the volatile emission of: (1) clean plants (CK); (2) *A. philoxeroides* plants with mechanical damage treatment (MD); and (3) *A. philoxeroides* plants infested with *A. hygrophila* 1st, 2nd, and 3rd larvae and female and male adults. A total of 97 volatiles were recorded, of which 5 occurred consistently in all treatments, while 61 volatiles were only observed in *A. philoxeroides* infested by *A. hygrophila*, such as trans-nerolidol, (E)-β-farnesene, and (3E,7E)-4,8,12-trimethyltrideca-1,3,7,11-tetraene (E, E-TMTT), etc. Among the 97 volatile compounds, 37 compounds belong to alkenes, 29 compounds belong to alkanes, and there were 8 esters, 8 alcohols and 6 ketones. Orthogonal partial least squares-discrimination analysis (OPLS-DA) showed that the different treatments were separated from each other, especially insect feeding from CK and MD treatments, and 19 volatiles contributed most to the separation among the treatments, with variable importance for the projection (VIP) values > 1. Our findings indicated that the alligatorweed plants could be induced to release volatiles by different stages of *A. hygrophila*, and the volatile compounds released differ quantitatively and qualitatively. The results from this study laid an important foundation for using volatile organic compounds (VOCs) and HIPVs of alligatorweed to improve the control effect of *A. hygrophila* on *A. philoxeroides*.

## 1. Introduction

Volatile organic compounds (VOCs) are the most important pathway through which plants emit signals to the environment [1]. VOCs play an important role in the vegetative relationship at plant-herbivorous insect-natural enemy [2,3,4]. In the long-term co-evolution of plants and herbivorous insects, plants gradually form an inductive defense response when they are threatened by herbivorous insects; that is, when plants are attacked by herbivorous insects, pest-induced VOCs will be released, which are called herbivore-induced plant volatiles (HIPVs) [5,6,7]. HIPV compounds can protect against herbivores directly or indirectly by enhancing the defense response, regulating insect behavior, and sending “early warning signals” to neighboring plants [8,9,10,11]. In addition, when plants are threatened by herbivorous insects, a volatile chemical signal, synomone, can be released to lure the natural enemies of herbivorous insects for indirect defense [12,13].

The sources of volatiles include mechanical damage caused by feeding insects and the induction of chemical attractants in insects. The types of plant volatiles induced by insect pests mainly include green leaf volatiles (GLVs), terpenoids, nitrogen-containing compounds, aldehydes, alcohols, ketones, ethers, and carboxylic acids [14,15]. The volatiles directly affects host selection, oviposition and courtship behavior in herbivores [16], while these behaviors of natural enemies would also affect their control effects on weeds. However, the current research on plant volatiles induced by pests mainly focuses on the relationship among crops, herbivorous pests and natural enemies of pests [17,18,19], and there have been few reports on VOC and HIPV components in invasive weeds affected by feeding of natural enemies for biological control.

*Alternanthera philoxeroides* (Mart.) Griseb is an important invasive weed native to South America [20]. *Agasicles hygrophila* Selman and Vogt (Coleoptera: Chrysomelidae) is a monophagous natural enemy and an effective biocontrol agent for *A. philoxeroides*. In the 1960s, *A. hygrophila* was first released to control *A. philoxeroides* in the southeastern United States, which was regarded as the first successful aquatic weed biological control program in the world [21,22]. In 1986, *A. hygrophila* was introduced into China, and 39 plants from 21 families were tested to confirm the specialism of *A. hygrophila* [23]. Specialists (i.e., monophagous and oligophagous insects) subsist on one or a few plants from the same family [24]. Studies on host specialization and adaptation have revealed that plant volatiles play important roles in host selection processes in herbivorous insects [25,26,27,28]. Li et al. demonstrated that the specialist beetle *A. hygrophila* adults used two common plant volatiles, (E)-4,8-dimethyl-1,3,7-nonatriene and (Z)-3-hexenol, for host discrimination [26]. However, little is known about *A. philoxeroides* responses to the feeding of *A. hygrophila* in different stages. Recognizing VOCs from *A. philoxeroides* will help to find new ways to manage this important invasive weed.

In the current study, we analyzed HIPVs emitted by undamaged and damaged *A. philoxeroides* (including mechanically damaged and *A. hygrophila*-infested) and identified the changes in emission profiles. Additionally, we also discussed the possible roles of these volatiles in plant defense, host localization, or natural enemy attraction. The results may help us to improve the control effect of *A. hygrophila* by using the volatiles of *A. philoxeroides*.

## 2. Materials and Methods

### 2.1. Plant and Insects

*Alternanthera philoxeroides* were grown in the greenhouses of Institute of Plant Protection, Fujian Academy of Agricultural Sciences (IPP, FAAS). *Agasicles hygrophila* were reared in the controlled laboratory of IPP, FAAS. Here, 1st, 2nd, and 3rd instar larvae, as well as female and male adults used in the experiments, were kept in climate cabinets (26 ± 2 °C, 70 ± 5% R.H., L14: D10) in plastic cases (18 × 11.5 × 7 cm) containing *A. philoxeroides* plants, respectively.

### 2.2. Plant Treatments

To characterize the differences in plant volatiles released in response to attack by mechanical damage and *A. hygrophila*, we collected headspace volatiles of *A. philoxeroides* plants subjected to different treatments. Plants were subjected to the three following treatments: (1) clean plants (CK), i.e., without herbivory and damaged; (2) mechanically damaged plants (MD); (3) *A. hygrophila*-infested plants, i.e., 1st instar larvae (1L), 2nd instar larvae (2L), 3rd instar larvae (3L), female adults (Female), and male adults (Male), respectively. *A. philoxeroides* stems (6–8 leaves and 20–25 cm in height) were used in the experiments and were placed in separate vials (d = 2.5 cm, h = 25 cm).

Mechanically damaged plants: 24 h before the volatiles was collected, *A. philoxeroides* leaves were cut with carborundum, a damaged spot of about 1 × 1 cm was created on each leaf, and then they were placed in separate vials.

*A. hygrophila*-infested plants: After 6 h of starvation, 6 individuals of 1st instar larvae, 2nd instar larvae, 3rd instar larvae, female adults, and male adults were released into each vial for 24 h, respectively, and then the insects and their feces were removed before collecting volatiles.

Clean plants, mechanically treated plants and herbivore-infested were kept in separate controlled chambers (26 ± 2 °C, 70 ± 5% R.H., L14: D10). All the samples were prepared in triplicate.

### 2.3. Collection of Plant Volatiles

After 24 h of mechanical damage treatment, volatiles were collected. The above *A. hygrophila*-infested plant was put into a new vial and kept for 24 h, respectively. Volatiles from healthy plants, mechanically damaged plants and *A. hygrophila*-infested plants were collected. After 24 h, the sample in the vial was equilibrated at 40 °C for 10 min in a water bath, respectively. After equilibration, the solid phase microextraction (SPME) was exposed to the headspace of the vial for 30 min, after which the SPME was inserted into a GC-MS (Shimadzu Corporation, Kyoto, Japan) desorption for 2 min for analysis.

### 2.4. Analysis of Plant Volatiles by GC-MS/MS

Volatile analysis was done with reference to the method described previously with some modifications [29]. GC-MS analysis was performed on the Nexis GC-2030 (GC) coupled with a QP2020NX mass spectrometer (Shimadzu Corporation). Helium was used as the carrier gas at a flow rate of 1 mL/ min in a splitless injection and a velocity of 36.3 cm/s. Volatile compounds were separated using a Rxi-5Sil MS (30 m × 0.25 mm, 0.25 μm) under the following conditions: the starting temperature of 50 °C was held for 2 min, followed by an increase from 50 to 180 °C at a rate of 5 °C/min, and then an increase to 280 °C at a rate of 20 °C/min, where the temperature was maintained until the procedure was manually stopped (a total of 35 min). The injector temperature was 200 °C, and the interface temperature was 280 °C. The mass spectrometric detector operated in the scan mode, and the m/z range was from 35 to 550. Compound identification was performed using the data system library (NIST 17-1, NIST 17-2, NIST 17s, FFNSC 1.2).

Volatile compounds were identified initially by comparing the mass spectra of the samples with the data system library (NIST 17-1, NIST 17-2, NIST 17s, FFNSC 1.2) and the retention index (RI). The compounds with a similarity index (SI) < 80 were deleted. The peak area normalization method was used to calculate the relative contents of each volatile compound.

### 2.5. Statistical Analysis

Orthogonal partial least squares-discrimination analysis (OPLS-DA) of the volatile compounds was performed using soft independent modelling of class analogies (SIMCA, Version 13.0.0.0, Umetrics AB, Umea, Sweden). The variable importance in the projection (VIP) value generated from OPLS-DA is often used to quantify the contribution of each material to the classification. The larger the VIP value is, the greater the contribution of the volatile compounds to discriminate the different groups. Therefore, the VIP value is usually taken as one of the important evaluation indexes. A heat map was obtained using OriginPro 2018C (SR1 b9.5.1.195, OriginLab Corporation, Northamnton, MA, USA).

## 3. Results

### 3.1. Volatile Compounds A. philoxeroides in Leaves Infested with A. hygrophila

Ninety-seven volatile substances were identified in *A. philoxeroides* of different treatments (Table 1). Nineteen compounds were identified from the clean plants (CK) of *A. philoxeroides*, mainly (E)-4,8-dimethylnona-1,3,7-triene (DMNT), heneicosane, trans-α-bergamotene and naphthalene. There were 26 volatile compounds in mechanically treated plants (MD), mainly heneicosane, tetracosane, cis-α-bergamotene, and (+)-β-cedrene. There were 32, 32, and 50 compounds, respectively, in *A. philoxeroides* infested by 1st, 2nd, and 3rd larvae *A. hygrophila*, mainly (E)-4,8-dimethylnona-1,3,7-triene, 2-ethenyl-1,1-dimethyl-3-methylene-cyclohexane and (E)-β-farnesene. The number of volatile compounds detected in *A. philoxeroides* leaves infested with female adults and male adults was 41 and 40, respectively, mainly including (E)-4,8-dimethylnona-1,3,7-triene (DMNT), trans-nerolidol, and 1-tridecene.

Among them, 5 compounds were detected in all treatments, and these compounds were identified as α-cedrene, 4,6-dimethyl-dodecane, eicosane, 2-ethylhexyl tert-butyl-ether, and heneicosane. Furthermore, 1 and 7 compounds were only found in the clean plants and the mechanically treated plants, respectively, and 61 volatiles were only observed in *A. philoxeroides* infested by *A. hygrophila*, which were absent in CK and MD treatments, and (3E,7E)-4,8,12-trimethyltrideca-1,3,7,11-tetraene (E, E-TMTT), trans-nerolidol, and (E)-β-farnesene were detected in the treatments with *A. hygrophila*.

**Figure 1:**
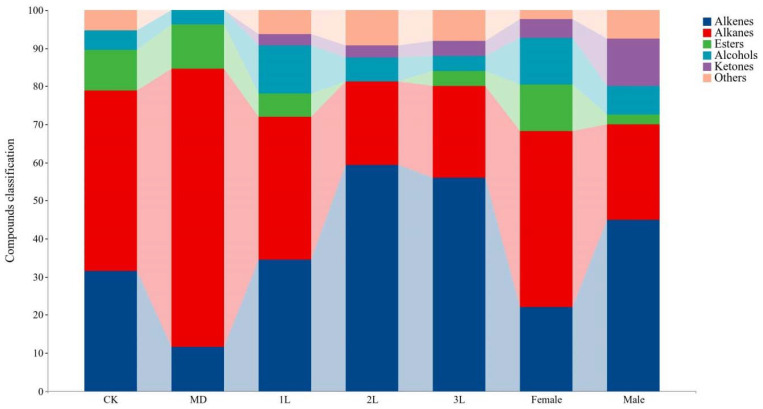
Variation in volatile compounds classification of the *A. philoxeroides* treated with clean plants (CK), mechanical damaged (MD), infested by 1st instar (1L), 2nd instar (2L), 3rd instar (3L), females and males of *A. hygrophila*.

Among the 97 volatile compounds, 37 compounds (38.14%) belong to alkenes, 29 compounds (29.90%) belong to alkanes, and there were 8 esters (8.25%), 8 alcohols (8.25%) and 6 ketones (6.19%) (Figure 1). In clean plants, volatile compounds included alkenes (6 compounds), alkanes (9 compounds), esters (2 compounds), alcohols (1 compound), and others (1 compound). The profile of the volatiles in mechanical treatments was as follows: alkenes (3 compounds), alkanes (19 compounds), esters (3 compounds), and alcohols (1 compound) (Figure 1). In the *A. philoxeroides* infested by 1st instar (1L), 2nd instar (2L), 3rd instar (3L), females and males of *A. hygrophila*, volatile compounds included alkenes (11, 19, 28, 9, 18 compounds), alkanes (12, 7, 12, 19, 10 compounds), esters (2, 0, 2, 5, and 1 compounds), alcohols (4, 2, 2, 5, and 3 compounds), ketones (1, 1, 2, 1, and 5 compounds), and others (2, 3, 4, 1, and 3 compounds), respectively.

### 3.2. OPLS-DA Analysis of Volatile Components in Leaves

**Figure 2:**
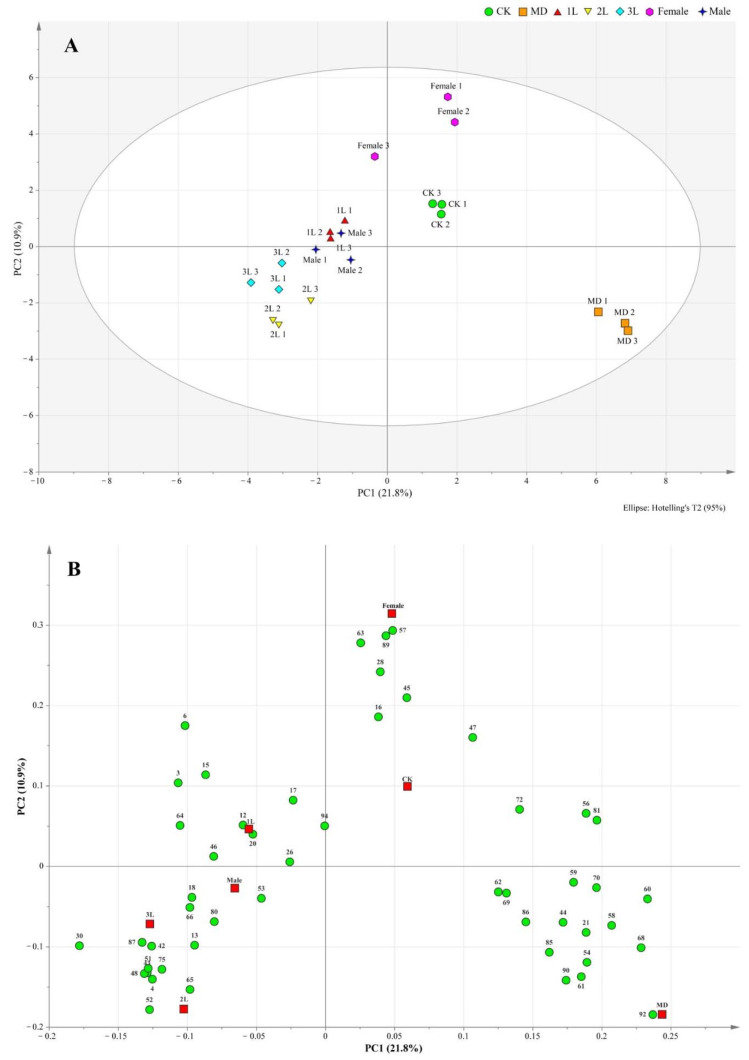
Score scatter plot (**A**) and loading scatter plot (**B**) of orthogonal partial least squares-discrimination analysis (OPLS-DA) based on headspace composition of *A. philoxeroides* treated with clean plants (CK), mechanically damaged (MD), and infested by 1st instar (1L), 2nd instar (2L), 3rd instar (3L), females and males of *A. hygrophila*. For compound identity in relation to the numbering in the loading plot, please refer to Table 1.

Projections to latent structures-orthogonal partial least-squares discrimination analysis (OPLS-DA) of all treatments together are presented in Figure 1, where the different treatments were separated from each other, especially insect feeding from CK and MD treatments (Figure 2A).

The correlations among the contributions of these compounds with the different treatments were clearly visible from the loading scatter plot (Figure 2B).

**Figure 3:**
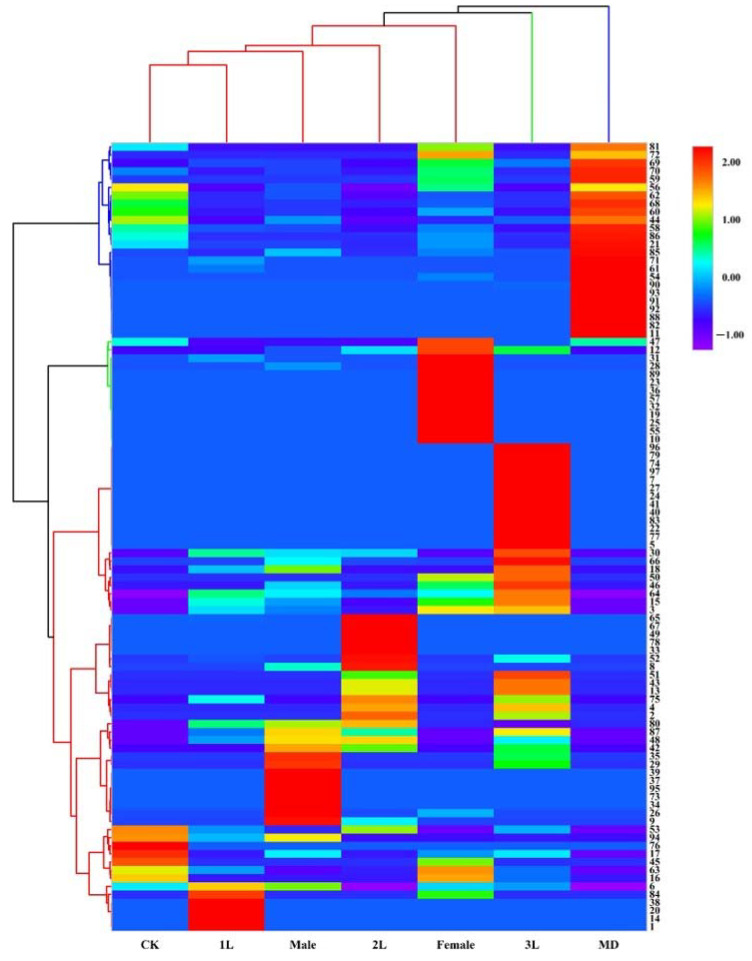
Heat map analysis of volatile compounds of *A. philoxeroides* treated with clean plants (CK), mechanically damaged (MD), and infested by 1st instar (1L), 2nd instar (2L), 3rd instar (3L), females and males of *A. hygrophila*.

The loading plot shows the distribution of volatile components corresponding to the distribution and location of sample points in the score plot. Among all the volatiles in this study, 19 contributed the most to the separation among the treatments, with variable importance for the projection (VIP) values >1 (Table 2). These compounds included 6, 15, 17, 20, 26, 30, 42, 43, 45, 48, 51, 52, 57, 63, 65, 66, 89, 92, 94. Heat map analysis showed that the volatile compounds of *A. philoxeroides* were well differentiated in different treatments (Figure 3).

## 4. Discussion

When plants are subjected to herbivorous insect stress, they may release different quantities and species of volatiles from those released by healthy plants and even resynthesize and/or release more stressed VOCs [30,31,32]. This study found that *A. philoxeroides* damaged mechanically and infested with *A. hygrophila* released some new VOCs, such as new volatiles in the plant after mechanical damage, including 5-methyl-5-propyl-nonane, 7-epi-sesquithujene, 4-methyl-tetradecane, and tetratetracontane, and volatiles that appeared after being infested by *A. hygrophila*, including (+)-longifolene, E, E-TMTT, trans-nerolidol, and (E)-β-farnesene. Cui et al. reported that compared with healthy plants and mechanically damaged plants, feeding-damaged plants released unique chemicals, including eucalyptol, phytol, and β-ocimene [33]. Feeding by *Tuta absoluta* (Meyrick) (Lepidoptera: Gelechiidae) and *Bemisia tabaci* (Gennadius) (Hemiptera: Aleyrodidae) induced quantitatively and qualitatively different HIPV blends from tomato plants [34]. This could be due to the direct or indirect defense response of host plants to regulate the behavior of herbivorous insects by adjusting the compounds of volatiles after being fed on by herbivorous insects.

Some volatiles also exist in healthy plants themselves, but the release amount increases significantly after being fed on by insects, which is also called HIPVs [35,36]. The increased HIPVs emitted from cotton plants damaged by *Agrotis segetum* (Lepidoptera: Noctuidae) larvae mainly consisted of linalool, β-caryophyllene, humulene, tetradecane, and hexadecane. [37]. In this study, it was found that the plants fed on by *A. hygrophila* released more (E)-4, 8-dimethylnona-1,3,7-triene (DMNT), α-farnesene, amd 8-hexyl-pentadecane (Table 1). These HIPVs play important roles in host plants’ resistance to herbivorous insect feeding [32]. The OPLS-DA analysis identified that 19 major volatile compounds played major roles in discriminating the plants fed by *A. hygrophila*, mechanically damage plants and healthy plants, such as (3E)-4, 8-dimethyl-1,3, 7-nontriene, copaene, caryophyllene, trans-nerolidol, cedarol and β-cedarene. These volatile compounds are closely related to the defense of host plants [38], regulation of insect behavior [18,39] and attraction of insect enemies [4,35].

Host localization and recognition by herbivorous insects depend on specific volatiles released by host plants [18,40]. Jacobi et al. found that *Dichelops furcatus* (Hemiptera: Pentatomidae) could distinguish hosts according to volatiles released by different varieties of the same hosts, and linalool was the main clue for their localization [16]. Li et al. demonstrated that the accurate localization of *A. hygrophila* was based on DMNT emitted by *A. philoxeroides*, and the effect of this compound on host selection preference could be effectively exploited in biological control [28]. In our study, the relative contents of DMNT emitted from *A. philoxeroides* infested by *A. hygrophila* were significantly higher than that of the clean plants. It is indicated that DMNT is an important clue in the localization and recognition of host plants of *A. hygrophila*.

Plant volatiles not only affect the behavior of herbivorous insects but also attract the natural enemies of herbivorous insects, especially the HIPVs [4]. The HIPVs released by *Tibraca limbativentris* Stål (Heteroptera: Pentatomidae) and *Glyphepomis spinosa* Campos et Grazia (Heteroptera: Pentatomidae) feeding on rice are attractive to the natural enemy of the rice pest, *Telenomus podisi* Ashmead (Hymenoptera: Platygastridae) [41]. Our results showed that trans-nerolidol, cis-3-hexenyl benzoate, and α-farnesene all appeared after being fed on by *A. hygrophila*. Numerous studies showed that terpenoids and GLVs, such as trans-nerolidol, cis-3-hexenoacetate, trans-β-farnesene and jasmonone, play major roles in the attraction of natural enemies [35,42]. However, no studies have reported the natural enemies of *A. hygrophila*, which may be further studied on this basis.

The emitting of volatiles by insects feeding on host plants is a complex process. An understanding of volatiles of *A. philoxeroides* infested by different stages *A. hygrophila* is needed for developing alligatorweed management strategies based on semiochemicals. The results of this study showed that new volatile compounds, such as (+)-longifolene, E, E-TMTT, trans-nerolidol, and (E)-β-farnesene, were produced in *A. philoxeroides* after being fed on by *A. hygrophila*. At the same time, the contents of some volatiles increased, such as (E)-4, 8-dimethylnona-1,3,7-triene (DMNT), α-farnesene, and so on. Among them, DMNT is an important substance for the host plant localization and recognition of *A. hygrophila*, while trans-nerolidol and (E)-β-farnesene play important roles in natural enemy attraction. In the following studies, attention should be paid to the application of VOCs and HIPVs in the population collection and field population migration and monitoring of *A. hygrophila*, so as to improve the prevention and biocontrol of *A. hygrophila* on *A. philoxeroides*.

## Figures and Tables

**Table 1:** Volatile components in healthy, mechanical damaged and *A. hygrophila* feeding of *A. philoxeroides*.

NO.	Compound	RI	CAS	Relative Content (%)						
				CK	MD	1L	2L	3L	Female	Male
1	(+)-Longifolene	1497	61262-67-7	-	-	0.22 ± 0.07	-	-	-	-
2	(1R,2S,6S,7S,8S)-8-Isopropyl-1-methyl-3-methylenetricyclo [4.4.0.02,7]decane-rel-	1431	18252-44-3	-	-	-	0.10 ± 0.03	0.07 ± 0.02	-	-
3	(3E,7E)-4,8,12-Trimethyltrideca-1,3,7,11-tetraene (E, E-TMTT)	1573	62235-6-7	-	-	1.34 ± 0.50	0.38 ± 0.12	2.89 ± 1.38	2.68 ± 1.50	0.89 ± 0.55
4	(4S,4aR,6R)-4,4a-Dimethyl-6-(prop-1-en-2-yl)-1,2,3,4,4a,5,6,7-octahydronaphthalene	1472	54868-40-5	-	-	-	0.75 ± 0.50	0.71 ± 0.32	-	-
5	(E)-1-Methyl-4-(6-methylhept-5-en-2-ylidene) cyclohex-1-ene	1596	53585-13-0	-	-	0.22 ± 0.77	0.12 ± 0.08	0.37 ± 0.05	0.19 ± 0.06	0.18 ± 0.06
6	(E)-4,8-Dimethylnona-1,3,7-triene (DMNT)	1106	19945-61-0	-	-	-	-	0.10 ± 0.05	-	-
7	α-Farnesene	1504	502-61-4	20.32 ± 2.27	-	78.64 ± 1.93	1.32 ± 0.42	34.34 ± 5.74	39.88 ± 3.67	65.80 ± 10.09
8	α-Guaiene	1497	3691-12-1	-	-	-	-	0.09 ± 0.04	-	-
9	β-Ocimene	1034	13877-91-3	-	-	-	0.31 ± 0.27	-	-	0.10 ± 0.09
10	1,2-Benzenedicarboxylic acid, bis(2-methylpropyl) ester	1861	84-69-5	-	-	-	0.28 ± 0.25	-	-	1.09 ± 0.96
11	1,2-Benzenedicarboxylic acid, butyl 2-ethylhexyl ester	1956	85-69-8	0.99 ± 0.86	-	0.30 ± 0.07	0.75 ± 0.30	0.34 ± 0.11	-	0.13 ± 0.11
12	1,3,6-Octatriene, 3,7-dimethyl-, (Z)-	1034	3338-55-4	-	-	-	-	-	0.09 ± 0.03	-
13	1,3-Cyclopentadiene, 5,5-dimethyl-2-propyl-	1170	878270-08-7	-	0.62 ± 0.21	-	-	-	-	-
14	1,4-Dimethyl-7-(prop-1-en-2-yl) decahydroazulen-4-ol	1497	21698-41-9	-	-	-	0.96 ± 0.22	1.48 ± 0.49	2.84 ± 0.50	0.35 ± 0.12
15	trans-Nerolidol	1561	40716-66-3	-	-	-	0.13 ± 0.12	0.17 ± 0.06	-	-
16	11-Methyltricosane	1749	27538-41-6	-	-	0.13 ± 0.12	-	-	-	-
17	β-Cedrene	1425	546-28-1	-	-	2.35 ± 0.70	0.31 ± 0.27	1.80 ± 1.20	3.22 ± 0.94	1.56 ± 0.94
18	1H-Cyclopropa[a]naphthalene, decahydro-1,1,3a-trimethyl-7-methylene-, [1aS-(1aα,3aα,7aβ,7bα)]-	1497	20071-49-2	1.86 ± 0.27	-	-	-	3.20 ± 1.58	0.87 ± 0.29	-
19	1-Tridecene	1083	2437-56-1	7.11 ± 2.25	7.81 ± 0.55	0.93 ± 0.17	0.65 ± 0.07	2.08 ± 0.58	1.43 ± 0.69	2.72 ± 1.65
20	2,2,4-Trimethyl-1,3-pentanediol diisobutyrate	1586	6846-50-0	1.81 ± 0.60	-	0.16 ± 0.05	0.16 ± 0.01	0.60 ± 1.79	0.43 ± 0.25	0.62 ± 0.31
21	2,6,10-Trimethyltridecane	1704	3891-99-4	-	-	0.15 ± 0.13	-	0.51 ± 0.17	-	0.34 ± 0.11
22	2,6-Dimethyl-1,3,5,7-octatetraene, E,E-	1124	460-1-5	-	-	-	-	-	4.29 ± 1.77	-
23	2-Hexadecanol	1473	14852-31-4	-	-	0.47 ± 0.02	-	-	-	-
24	2-Isopropenyl-4a,8-dimethyl-1,2,3,4,4a,5,6,8a-octahydronaphthalene	1497	207297-57-2	-	3.72 ± 1.03	0.17 ± 0.08	-	-	0.57 ± 0.23	0.17 ± 0.06
25	2-Isopropyl-5-methyl-1-heptanol	1316	91337-7-4	-	-	-	-	0.18 ± 0.04	-	-
26	2-Nonadecanone	1464	629-66-3	-	-	-	-	-	0.50 ± 0.17	-
27	2-Pentadecanone, 6,10,14-trimethyl-	1845	502-69-2	-	-	-	-	0.17 ± 0.06	-	-
28	2-Undecanone, 6,10-dimethyl-	1464	1604-34-8	-	-	-	-	-	0.16 ± 0.07	-
29	3,7-Nonadien-2-ol, 4,8-dimethyl-	1084	67845-50-5	-	-	-	-	-	0.06 ± 0.05	0.39 ± 0.26
30	3-Buten-2-one, 4-(2,6,6-trimethyl-1-cyclohexen-1-yl)-	1478	14901-7-6	-	-	-	-	0.39 ± 0.46	-	-
31	3-Ethyl-3-methylheptane	1043	17302-1-1	-	-	-	-	-	1.28 ± 0.38	0.12 ± 0.04
32	cis-3-hexenyl benzoate	1571	25152-85-6	-	-	-	-	0.72 ± 0.24	-	1.42 ± 0.47
33	3-Tetradecen-5-yne, (E)-	1215	74744-44-8	-	-	0.37 ± 0.03	0.27 ± 0.17	0.77 ± 0.12	-	0.27 ± 0.09
34	3-Tetradecene, (E)-	1083	41446-68-8	-	-	0.15 ± 0.05	-	-	1.22 ± 1.08	-
35	Azulene	1180	275-51-4	-	-	-	-	-	3.50 ± 1.95	-
36	Benzene, 1,3-bis(1-methylethyl)-	1641	99-62-7	-	-	-	0.51 ± 0.33	-	-	-
37	Benzene, 1-ethyl-3,5-dimethyl-	1144	934-74-7	-	-	-	-	-	-	2.55 ± 0.25
38	Benzene, 2-ethyl-1,4-dimethyl-	1144	1758-88-9	-	-	-	-	0.09 ± 0.02	-	0.21 ± 0.18
39	Bicyclo [3.1.1]hept-2-en-6-one, 2,7,7-trimethyl-	1170	473-6-3	-	-	-	-	-	0.30 ± 0.10	-
40	Bicyclo [5.2.0]nonane, 2-methylene-4,8,8-trimethyl-4-vinyl-	1490	242794-76-9	-	-	-	-	-	-	0.61 ± 0.20
41	(-)-Isocaryophyllene	1599	118-65-0	-	-	0.18 ± 0.06	-	-	-	-
42	β-Bisabolene	1508	495-61-4	-	-	-	-	-	-	0.15 ± 0.14
43	Caryophyllene	1421	87-44-5	-	-	-	-	0.71 ± 0.34	-	-
44	α-Cedrene	1417	469-61-4	-	-	-	-	0.10 ± 0.06	-	-
45	Cedrol	1611	77-53-2	-	-	-	0.21 ± 0.12	0.18 ± 0.09	-	0.29 ± 0.08
46	β-Chamigrene	1421	18431-82-9	-	-	-	0.24 ± 0.13	0.31 ± 0.16	-	-
47	cis-α-Bergamotene	1434	18252-46-5	1.72 ± 0.53	-	-	-	-	0.95 ± 0.20	-
48	Copaene	1376	3856-25-5	-	-	-	-	0.32 ± 0.11	0.15 ± 0.13	0.09 ± 0.08
49	Cycloheptane, 4-methylene-1-methyl-2-(2-methyl-1-propen-1-yl)-1-vinyl-	1497	826337-63-7	3.60 ± 2.62	3.28 ± 2.89	-	-	1.12 ± 0.1	7.25 ± 4.42	0.22 ± 0.02
50	Cyclohexane, 1,1-dimethyl-2-propyl-	1084	81983-71-3	-	-	0.32 ± 0.29	0.93 ± 0.13	0.45 ± 0.72	-	0.9 ± 0.43
51	Cyclohexane, 1-ethenyl-1-methyl-2,4-bis(1-methylethenyl)-, [1S-(1α,2β,4β)]-	1390	515-13-9	-	-	-	0.12 ± 0.04	-	-	-
52	Cyclohexane, 2-ethenyl-1,1-dimethyl-3-methylene-	1084	95452-8-7	-	-	-	-	2.07 ± 0.69	1.48 ± 1.31	-
53	Cyclosativene	1369	22469-52-9	-	-	-	0.10 ± 0.05	0.34 ± 0.11	-	-
54	Decane, 1-iodo-	1694	2050-77-3	-	-	2.57 ± 0.83	77.41 ± 7.02	22.79 ± 7.34	-	0.94 ± 0.83
55	Decane, 3,7-dimethyl-	1091	17312-54-8	-	2.23 ± 1.14	-	-	-	0.15 ± 0.05	-
56	Dibutyl phthalate	1956	84-74-2	-	-	-	-	-	0.12 ± 0.01	-
57	Diethyl Phthalate	1585	84-66-2	6.17 ± 0.52	3.20 ± 0.78	0.29 ± 0.09	-	0.27 ± 2.02	2.11 ± 0.38	0.83 ± 0.56
58	Docosane	1703	629-97-0	-	-	-	-	-	0.95 ± 0.21	-
59	Dodecane, 2,6,11-trimethyl-	1092	31295-56-4	1.44 ± 0.30	3.10 ± 0.68	0.34 ± 0.1	-	-	0.54 ± 0.16	0.31 ± 0.28
60	Dodecane, 4,6-dimethyl-	1320	61141-72-8	-	1.86 ± 1.09	-	-	-	0.78 ± 0.24	-
61	Dodecane, 4-methyl-	1092	6117-97-1	6.93 ± 0.63	6.03 ± 0.47	0.36 ± 0.11	0.16 ± 0.03	0.28 ± 0.23	1.63 ± 0.48	0.67 ± 0.48
62	Eicosane	1698	112-95-8	-	-	0.47 ± 0.13	-	-	-	-
63	Ether, 2-ethylhexyl tert-butyl	1027	83704-3-4	8.71 ± 0.51	8.81 ± 4.31	0.75 ± 0.09	0.09 ± 0.08	0.80 ± 0.27	1.25 ± 0.20	1.25 ± 0.10
64	(E)-β-Farnesene	1452	18794-84-8	6.55 ± 4.11	7.2 ± 2.4	3.21 ± 0.86	1.16 ± 0.17	2.42 ± 0.74	10.01 ± 2.31	0.19 ± 0.17
65	cis,cis-Farnesol	1555	16106-95-9	-	-	0.08 ± 0.01	-	-	-	-
66	Furan, 3-(4,8-dimethyl-3,7-nonadienyl)-, (E)-	1573	23262-34-2	-	-	-	7.04 ± 2.77	9.33 ± 4.97	-	2.52 ± 0.84
67	α-Gurjunene	1500	489-40-7	-	-	-	0.08 ± 0.02	-	-	-
68	Heneicosane	1626	629-94-7	11.57 ± 0.81	14.06 ± 2.07	0.24 ± 0.06	0.14 ± 0.07	0.38 ± 0.07	1.25 ± 0.38	0.54 ± 0.04
69	Heptadecane	1320	629-78-7	-	1.66 ± 1.46	0.18 ± 0.04	-	0.29 ± 0.35	0.83 ± 0.27	0.15 ± 0.05
70	Hexadecane, 2,6,10,14-tetramethyl-	1807	638-36-8	0.42 ± 0.06	2.62 ± 0.54	-	-	0.07 ± 0.04	1.16 ± 0.27	0.16 ± 0.05
71	Hexadecane, 2,6,11,15-tetramethyl-	1534	504-44-9	-	1.43 ± 1.26	0.16 ± 0.05	-	-	-	-
72	Hexatriacontane	1839	630-6-8	-	0.53 ± 0.07	-	-	-	0.56 ± 0.17	-
73	α-Himachalene	1420	3853-83-6	-	-	-	-	-	-	0.14 ± 0.05
74	α-Humulene	1457	6753-98-6	-	-	-	-	0.08 ± 0.04	-	-
75	isoledene	1499	95910-36-4	-	-	0.11 ± 0.01	0.27 ± 0.08	0.20 ± 0.07	-	-
76	Naphthalene	1179	91-20-3	9.24 ± 1.97	-	-	-	-	-	-
77	Naphthalene, 1,2,3,4,4a,5,6,8a-octahydro-4a,8-dimethyl-2-(1-methylethenyl)-, [2R-(2α,4aα,8aβ)]-	1497	473-13-2	-	-	-	-	0.2 ± 0.17	-	-
78	Naphthalene, 1,2,3,4,4a,5,6,8a-octahydro-7-methyl-4-methylene-1-(1-methylethyl)-, (1α,4aβ,8aα)-	1476	39029-41-9	-	-	-	0.32 ± 0.22	-	-	-
79	Naphthalene, 1,2,3,5,6,7,8,8a-octahydro-1,8a-dimethyl-7-(1-methylethenyl)-, [1R-(1α,7β,8aα)]-	1494	4630-7-3	-	-	-	-	0.08 ± 0.04	-	-
80	Nerolidol	1561	7212-44-4	-	-	0.34 ± 0.12	0.70 ± 0.45	1.12 ± 0.34	-	1.17 ± 0.45
81	Nonadecane	1534	629-92-5	-	0.56 ± 0.19	2.37 ± 0.79	3.91 ± 0.48	-	0.52 ± 0.06	3.26 ± 1.09
82	Nonane, 5-methyl-5-propyl-	1274	17312-75-3	0.94 ± 0.81	2.50 ± 1.11	-	-	-	1.78 ± 0.71	-
83	(E)-β-Ocimene	1034	3779-61-1	-	2.36 ± 2.08	-	-	-	-	-
84	Octadecane	1281	593-45-3	-	-	-	-	3.46 ± 1.13	-	-
85	Pentacosane	1911	629-99-2	-	-	0.20 ± 0.02	-	-	0.11 ± 0.04	-
86	Pentadecane, 8-hexyl-	1749	13475-75-7	0.38 ± 0.08	7.24 ± 1.42	-	0.11 ± 0.03	0.47 ± 0.05	0.87 ± 0.38	1.57 ± 0.33
87	β-Selinene	1490	17066-67-0	0.58 ± 0.42	1.43 ± 1.26	-	-	-	0.21 ± 0.06	-
88	7-epi-Sesquithujene	1434	159407-35-9	-	1.65 ± 0.52	-	-	-	-	-
89	Tetracontane	1807	4181-95-7	-	-	-	-	-	1.59 ± 0.26	-
90	Tetracosane	1749	646-31-1	-	10.5 ± 1.92	-	-	0.11 ± 0.45	-	-
91	Tetradecane, 4-methyl-	1321	25117-24-2	-	2.21 ± 0.74	-	-	-	-	-
92	Tetratetracontane	1910	7098-22-8	-	2.69 ± 0.22	-	-	-	-	-
93	Tetratriacontyl heptafluorobutyrate	1299	84461-48-3	-	0.70 ± 0.23	-	-	-	-	-
94	trans-α-Bergamotene	1433	13474-59-4	9.66 ± 2.14	-	1.64 ± 0.52	-	0.13 ± 0.04	-	4.56 ± 0.96
95	trans-β-Ionone	1478	79-77-6	-	-	-	-	-	-	0.52 ± 0.06
96	Tricyclo [3.1.0.0(2,4)]hexane, 3,6-diethyl-3,6-dimethyl-, trans-	1170	58987-01-2	-	-	-	-	0.36 ± 0.12	-	-
97	Z,Z,Z-4,6,9-Nonadecatriene	1287	89353-62-8	-	-	-	-	0.56 ± 0.17	-	-

Note: “-” means not detected. CK: clean plants, without herbivory and damaged; MD: mechanically damaged plants; 1L: *A. hygrophila*-infested plants by 1st instar larvae; 2L: *A. hygrophila*-infested plants by 2nd instar larvae; 3L: *A. hygrophila*-infested plants by 3rd instar larvae; Female: *A. hygrophila*-infested plants by female adults; and Male: *A. hygrophila*-infested plants by male adults.

**Table 2:** Values of variable importance in the projection (VIP) of volatile compounds for the corresponding OPLS-DA plots (VIP > 1).

NO.	Compounds	VIP
20	2,2,4-Trimethyl-1,3-pentanediol diisobutyrate	1.99458
89	Tetracontane	1.38855
65	cis,cis-Farnesol	1.38627
57	Diethyl Phthalate	1.36926
26	2-Nonadecanone	1.33858
52	Cyclohexane, 2-ethenyl-1,1-dimethyl-3-methylene-	1.32584
6	(E)-4,8-Dimethylnona-1,3,7-triene	1.31482
66	Furan, 3-(4,8-dimethyl-3,7-nonadienyl)-, (E)-	1.24214
94	trans-α-Bergamotene	1.19307
45	Cedrol	1.19001
17	β-Cedrene	1.14817
63	Ether, 2-ethylhexyl tert-butyl	1.13473
51	Cyclohexane, 1-ethenyl-1-methyl-2,4-bis(1-methylethenyl)-, [1S-(1α,2β,4β)]-	1.10913
42	β-Bisabolene	1.10461
30	3-Buten-2-one, 4-(2,6,6-trimethyl-1-cyclohexen-1-yl)-	1.07133
92	Tetratetracontane	1.05905
15	1,6,10-Dodecatrien-3-ol, 3,7,11-trimethyl-, (E)-	1.05126
48	Copaene	1.02700
43	Caryophyllene	1.01986

## Data Availability

Not applicable.
